# POLICIES AND PERSPECTIVES AROUND SEXUAL ACTIVITIES AMONG RESIDENTS WITH COGNITIVE IMPAIRMENT OR DEMENTIA IN LTC

**DOI:** 10.1093/geroni/igac059.1773

**Published:** 2022-12-20

**Authors:** Mijin Jeong, Sarah Jen

**Affiliations:** University of Kansas, Lawrence, Kansas, United States; University of Kansas, Lawrence, Kansas, United States

## Abstract

Many older adults remain sexually interested and active in later life. However, little is known about how sexual policies and practices in skilled-nursing facilities (SNF) address sexual activities of residents with cognitive impairment and dementia. This study seeks to identify the current sexual policies and staff’s perspectives related to residents with cognitive impairment or dementia in SNFs in Kansas. Online surveys and mailed surveys were distributed to administrators from all 364 SNFs in Kansas in June 2020. 60 long-term care facilities (16.5%) answered the survey. Of 60 survey respondents, 22 facilities (36.7%) have a policy addressing sexual expression and 19 of those policies (94.7%) address issues related to cognitive impairment, competency, or dementia. 77.4% had trained their staff on the impact on sexual expression for those with cognitive impairment or dementia once or more than once during the past year. 73.3% of administrators stated that their staff would respond differently to sexual expression among individuals with dementia or cognitive impairment compared to other residents, often noting issues related to consent and capacity. 55.2% reported any sexual expression among residents with dementia within the past year. Findings indicated that there is a lack of overall sexual policies, but those that exist are likely to address residents with cognitive impairment or dementia. Although there is evidence of training and attention to issues related to sexual expression in individuals with dementia or cognitive impairment, there is a need for further efforts to establish practice norms and policies around more complex or nuanced situations.

